# Multilevel predictors of ultra-processed food intake in Canadian preschoolers

**DOI:** 10.1038/s43856-026-01473-1

**Published:** 2026-03-06

**Authors:** Sara Mousavi, Zheng Hao Chen, Zihang Lu, Susana Santos, Mary R. L’Abbe, Meghan B. Azad, Piushkumar J. Mandhane, Theo J. Moraes, Padmaja Subbarao, Stuart E. Turvey, Jeffrey R. Brook, Kozeta Miliku

**Affiliations:** 1 Department of Nutritional Sciences, University of Toronto, Toronto, ON Canada; 2 Department of Public Health Sciences, Queen’s University, Kingston, ON Canada; 3 EPIUnitITR, Institute of Public Health of the University of Porto, University of Porto, Rua das Taipas, Porto, Portugal; 4 Department of Pediatrics and Child Health, University of Manitoba, Winnipeg, MB Canada; 5 Department of Pediatrics, University of Alberta, Edmonton, AB Canada; 6 Faculty of Medicine, UCSI University, Kuala Lumpur, Malaysia; 7 Department of Pediatrics, The Hospital for Sick Children, Toronto, ON Canada; 8 Department of Physiology, University of Toronto, Toronto, ON Canada; 9 Department of Medicine, McMaster University, Hamilton, ON Canada; 10 Dalla Lana School of Public Health, University of Toronto, Toronto, ON Canada; 11 Department of Pediatrics, BC Children’s Hospital, University of British Columbia, Vancouver, BC Canada

**Keywords:** Epidemiology, Paediatric research

## Abstract

**Background:**

Ultra-processed foods (UPF) dominate modern food systems and contribute significantly to early-life diets. However, the multilevel predictors of UPF consumption in early childhood, from family factors to neighbourhood environments, remain underexplored.

**Methods:**

We leveraged data from a subset of the Canadian CHILD Cohort Study (n = 2,411), to assess UPF intake in three-years-old children using the NOVA classification system. A machine-learning variable selection algorithm and mixed-effect models identified independent predictors of UPF spanning family behaviours to neighbourhood environments.

**Results:**

Here we show parental factors including prenatal maternal UPF intake (β = 2.8 % daily energy from UPF, [95%CI 2.3,3.2]) and greater paternal adherence to a Western-like dietary pattern (β = 1.1, [95%CI 0.6,1.6]) are associated with higher UPF intake. Other factors such as shorter breastfeeding duration, longer daily screen time, and having older siblings are also associated with a higher proportion of daily energy intake from UPF at three years of age (all p-values < 0.05). In contrast, children residing in neighbourhoods with better access to employment opportunities (β = –1.9, [95%CI –3.0,–0.9]) and higher density of fresh food markets (β = –2.0, [95%CI –3.4,–0.5]) are associated with lower proportion of daily energy intake from UPFs.

**Conclusions:**

These findings indicate that the early childhood UPF intake reflects the convergence of family behaviours and structural features of the built environment. Interventions to reduce UPF intake must go beyond individual food choice and address food systems design, including how the interrelated factors of daily time demands, travel distance requirements and public infrastructure constrain access to healthier options that shape children’s diet.

## Plain Language Summary

Ultra-processed foods now make up a large part of young children’s diets and are linked to poorer physical and mental health. However, it remains unclear why some children consume more ultra-processed foods than others. Using data from over 2,400 Canadian preschoolers, we examined how family habits and neighbourhood environments together shape children’s ultra-processed food intake. We found that children ate more ultra-processed foods when their parents consumed more of these foods, when screen time was higher, and when families lived in areas with limited access to fresh food markets or longer travel times to work. Altogether, these findings show that early-life diet is shaped by both family and the environment children grow up in. Effective policies must therefore go beyond individual choices to improve food environments and support healthy childhood development.

## Abstract (web summary)

Mousavi, Chen, Miliku et al. analyse dietary, family, and neighbourhood data from Canadian preschoolers to identify predictors of ultra-processed food intake. They find that higher intake is linked to parental diets, shorter breastfeeding, screen time, having older siblings, and poorer access to jobs and fresh food markets, among other factors.

## Introduction

Ultra-processed foods (UPF) have transformed global food systems, reshaping diets through industrial processing, extended shelf lives, and convenience-driven marketing^1,2^. These foods, often characterised by high levels of added sugars, sodium, saturated fats, and additives, are now the primary source of energy intake in many high-income countries, including Canada, which has some of the highest volume sales of UPF per capita^3–5^. Unlike whole foods or minimally processed foods (MPF), UPF are typically manufactured using ingredients not commonly used in home cooking and undergo a series of industrial processes that alter intrinsic food matrices^5^.

UPF not only displaces nutrient-rich MPF, but also contributes to the development of adverse health outcomes and comes with sustainability concerns^6,7^. In response, some countries have updated their dietary guidelines and implemented food system policies, such as taxes on sugar-sweetened beverages, front-of-package labelling to warn about nutrients of concern, and marketing restrictions to limit intakes of UPF^8^. However, these interventions focused on nutrient content, despite evidence from clinical trials suggesting that MPF results in better health outcomes than UPF, even when diets were matched nutritionally^9,10^. To advance policy approaches that explicitly address UPF, a deeper understanding of the factors driving their consumption is essential. These insights are especially critical in early life when eating patterns are first established and children are especially vulnerable to the food environments, thus enabling policy interventions to yield the most impactful long-term benefits^11^.

In Canada and the United States, nearly half of children’s daily energy intake is derived from UPF^3,12^. This poses great concern as most studies, including our work in Canada^13^, find that a greater UPF exposure is associated with a higher prevalence of overweight/obesity and cardiometabolic comorbidities in children and adolescents^14^. Early dietary exposures not only track into adulthood^15^, but are also shaped by intersecting influences of family behaviours, built environment, and social structures. Existing evidence at a national and global scale suggests socioeconomic status, male sex, physical inactivity, non-immigrant status and urbanisation may influence UPF intakes across the population^16–18^, but most studies remain cross-sectional and narrowly focused on individual-level characteristics. Although several studies have simultaneously examined social and environmental determinants of children’s consumption of foods high in sugar, salt, or fat, including sugar-sweetened beverages^19^, few have integrated parental behaviours, child characteristics, and neighbourhood-level environments altogether within an explicit UPF framework in early childhood^20–24^, a developmental period critical to long-term health and development.

In this study, we use a systems-based lens to identify multilevel predictors of UPF intake in three-year-old children in a large Canadian pregnancy cohort. By combining individual, familial, and neighbourhood-level data, including food retail density and job accessibility, we aim to clarify how home environments interact with the urban environment to influence children’s diets. Our findings suggest that independent factors, ranging from family (e.g., parental diet, having older siblings) and the built environment (e.g., accessibility to jobs), are associated with childhood UPF intake. These results address a central challenge in population health: how individual behaviours, family circumstances, and structural urban design jointly shape diet in children. By integrating multilevel data across these domains, we offer a systems-based lens that extends beyond nutrition policy into public health, urban planning, and social equity. Embedding an equity lens within these strategies is essential, as disadvantaged communities often face disproportionate barriers to accessing healthy foods and supportive environments, and may therefore benefit most from structural policy changes.

## Methods

### Study design and population

The CHILD Cohort Study is a prospective, population-based pregnancy cohort, with four sites across Canada: Vancouver, Edmonton, Manitoba (Winnipeg and Morden/Winkler), and Toronto. Pregnant women were recruited into the CHILD study between 2008 and 2012 (*N* = 3621). Among those recruited, 3454 mothers (95.4%) consented to continued participation and delivered singletons who met the eligibility criteria (gestational age greater than 34 weeks + 4 days and no congenital abnormalities). These mother–child dyads remained eligible for follow-up (Supplementary Fig. 1)^25,26^. For the current study, children were excluded if they had missing dietary intake data (*n* = 1021), more than 10% of the FFQ incomplete (*n* = 5), or implausible total energy intake values, defined as energy intake above or below 3 SDs from the z-log energy mean (*n* = 18). This resulted in a final study population of *n* = 2411 children at the age of three years (Supplementary Fig. 1).

### Ethics approval and consent to participate

This study was conducted according to the guidelines laid down in the Declaration of Helsinki and all procedures involving research study participants were approved by the Human Research Ethics Boards at University of Toronto, McMaster University and Universities of Manitoba, Alberta, and British Columbia and the Hospital for Sick Children. This study was approved by the REBs at University of Toronto (RIS# 47810). Caregivers provided written informed consent.

### Parental demographics, diet, and health

A combination of factors collected in the study from enrolment or the prenatal period (i.e., 18 weeks of gestational age) to the postnatal period (i.e., three months, six months, one year, three years) were examined to identify potential predictors.

Prenatal and postnatal questionnaires, completed by parents at multiple visits, were used to capture data related to demographics, diet, and health. These included the parents’ age at offspring’s birth (years), education at enrolment (completion of a post-secondary degree), and ethnicity at enrolment (Caucasian or Non-Caucasian). As a measure of socioeconomic status (SES), at the enrolment and three-year visits, parents were asked to rate their subjective evaluation of social status in the community and in Canada, ranging from 0 (lowest standing) to 10 (highest standing). Mother’s marital status (single (includes single, divorced, widowed) vs. married), annual family income (<$100,000; ≥$100,000; prefer not to say), percent of government sources contributing to income (%), and housing status (rent vs. own) were collected from the enrolment and the three-year visits^25,26^.

Prenatal parental diet was collected during early pregnancy at the enrolment visit. Prenatal maternal diet was assessed using a validated food frequency questionnaire (FFQ) comprised of 151 food items developed by the Fred Hutchinson Cancer Research Centre^27^. This FFQ has been used in multiple prospective cohort studies and captures the intake frequency of specific food items, allowing all 151 items to be classified according to the NOVA classification system. The energy contribution from the mother’s UPF intake was derived using the same method for calculating the child’s UPF consumption as detailed below. Meanwhile, prenatal paternal diet was assessed using a 15-item food screener. This screener was derived from the International Study of Asthma and Allergies in Childhood (ISAAC) Phase III environmental questionnaire^28,29^ and has been widely used in epidemiological research, although it does not provide item-level dietary detail required for NOVA classification. Therefore, paternal UPF intake could not be directly estimated. To characterise paternal diet, we applied principal component analysis to the food group screener responses to derive dietary patterns^30^. This data-driven approach identified two dietary patterns, interpreted as Western-like and prudent dietary patterns.

Paternal and maternal body mass index (BMI) were calculated from height and weight measurements taken during the prenatal or one-year visits. Prenatal parental health history for conditions including depression, diabetes, food allergies, and preeclampsia was collected through questionnaires at enrolment. Maternal stress was assessed using the Perceived Stress Scale (PSS-10) questionnaire at enrollment and three-year timepoints.

### Child predictors

Child characteristics were collected from birth to three years of age. Medical records at birth were used to collect information on child sex (male vs. female), birth weight (grams), gestational age (weeks), and delivery mode (cesarean section vs. vaginal). Child ethnicity was derived from parents’ ethnicity data (Caucasian vs. non-Caucasian). The duration of breastfeeding (months), and introduction of solid foods were collected via repeated questionnaires from birth to one-year visit, as well as whether the child was exclusively breastfed (yes vs. no) at 6 months^31^. Information on child health (i.e., wheezing, atopic dermatitis, asthma, food allergy and food sensitivity) was also collected via parental assessments, clinical tests and physician assessments from six-month to three-year visits. Child height and weight measurements at the one-year and the three-year visits were used to calculate BMI. Screentime at age three years (hours/day), having older siblings (yes vs. no), and time spent away from home (proxy of daycare attendance) at ages one and three years were also collected via questionnaires.

### Environmental predictors

Household smoking and the presence of pets at home at age one year, and residential area type (urban vs. rural) provided by census data at three years, were included in the analysis. An urban area was defined to have at least 1000 people with a density of 400 or more people per square kilometre, where rural regions encompass the territories outside an urban area^32^. We included study sites (Edmonton, Manitoba, Toronto, and Vancouver) and seasons at which dietary assessments were conducted in the analysis.

Neighbourhood food environment at three years of age was derived using the Canadian Food Environment Dataset (Can-FED) accessed through the Canadian Urban Environmental Health Research Consortium (CANUE)^33,34^. In Can-FED, environmental exposures are defined at the dissemination area (DA) level, the smallest standard geographic unit used by Statistics Canada^35^. These retail food access measures, calculated according to 3-km buffers around the population-weighted centroids of the DAs, were re-mapped to six-digit postal codes to enable linkage to CHILD home postal codes. The density of fruit and vegetable markets (i.e., stores that primarily sell fresh fruits and vegetables) was derived by summing up the total number of such markets per square km within each 3 km buffer area, representing the local neighbourhood food environment^33^. These food outlets do not include chain grocery stores that sell a combination of fresh and packaged food items^33^. Therefore, fruit and vegetable markets were used as a more targeted proxy for MPF neighbourhood accessibility than traditional chain grocery stores. However, in a sensitivity analysis, we also examined if neighbourhood accessibility to chain grocery stores was associated with child UPF intake. In the same buffer area, a fast-food restaurant mix was used, which was derived by calculating the proportion of the density of fast-food restaurants (i.e., eating places that typically sell pre-prepared or quickly prepared food) relative to fast-food and full-service restaurants combined^33^. Fast-food restaurants include chain fast-food brand name or restaurants with terms referring to fast-food (i.e., fries, burger, pizza etc)^33^. These categorical variables were grouped where ‘no accessibility’ to food outlets in the designated area is the reference category and is compared to ‘lowest to moderate density’ and ‘moderate to highest density’ of food outlets in the designated area^33^.

In addition, the competitive accessibility to employment opportunities via transit or car, as derived by CANUE and described in detail elsewhere^34,36^, was used to assess the effect of family’s proximity to employment on childhood diet. These continuous variables with a score ranging from 0 to 1 (where higher indicates higher competitive job accessibility) were dichotomised at the median (0.55 and 0.23 for job accessibility by car and transit, respectively) for ease of interpretation in multivariable regression models.

### Child dietary assessment

Dietary intake data were collected using a semiquantitative 112-item food frequency questionnaire (FFQ), completed by the caregiver at the three-year clinical visit. The FFQ was validated in a subset of the FAMILY (Family Atherosclerosis Monitoring in Early Life) study^37,38^. The daily energy and nutrient intakes for each food item were calculated and outlined in detail elsewhere^13^. All 112 food items were then classified into four groups according to the NOVA classification system: 1) *unprocessed or MPF*, 2) *processed culinary ingredients (PCI)*, 3) *processed foods (PF)*, and 4) *UPF*^5^. The dietary energy shares (%) contributed by UPF was calculated by dividing the energy intake from UPF by the total daily energy intake, then multiplied by 100.

### Statistics and reproducibility

Mean and standard deviation (SD) were used to describe normally distributed continuous variables, whereas median and interquartile range (IQR) were used to describe the non-normally distributed continuous variables. For categorical variables, n (%) were reported. Statistical analyses were conducted in three sequential steps. First, crude (unadjusted) linear regression analyses were performed to examine the direction of associations between individual parental, child, and environmental factors and UPF intake (% of total daily energy) at age three years. Second, all candidate predictor variables, regardless of statistical significance in crude analyses, were examined using a Deletion/Substitution/Addition (*partDSA*) variable selection algorithm^39^ to identify the most relevant predictors. Third, predictors selected by the partDSA algorithm were mutually adjusted in multivariable linear mixed-effects models to estimate independent associations with UPF intake. We used crude regression analyses to identify directions of individual correlates (parental behaviours, childhood characteristics, and the built environment) on UPF consumption (% of daily energy intake) at age three. Predictors of UPF consumption were identified using the Deletion/Substitution/Addition algorithm implemented via the *partDSA* package^39^. This widely used prediction algorithm partitions the covariate space to predict outcomes that are influenced by multiple variables^39^. Given that this algorithm relies on cross-validation, we ran 100 partDSA models per dataset across the 10 imputed datasets. In our study, multiple imputation was done in the subset of n = 2,411, after excluding participants with missing dietary intake data, incomplete FFQ, and energy intake outliers. Missing data, ranging from 0.12% for maternal gestational diabetes to 19.95% for prenatal paternal BMI (Supplementary Table 1), were imputed using the multivariate imputation by chained equations implemented via the *mice* package^40^, a common approach used in observational studies with mixed variable types^41^. Significant predictors were defined as variables identified in at least 5% of models^42^.

Furthermore, to identify the independent predictors of UPF consumption, we mutually adjusted all predictors identified in the partDSA algorithm in a linear regression model. Our intraclass correlation test indicates that around 7% of the variance for UPF is between study sites; therefore, we used a multivariable-adjusted linear mixed-effect model, with study sites as a random effect. To enable comparison between effect estimates for different predictors, we converted measurements into a comparable scale (per SD-increase). We assessed potential multicollinearity in our multivariable regression model by using correlation analysis and collinearity diagnostic testing (i.e., variance inflation factors (VIF)). All predictors included in the multivariable models had VIF values ranging from 1.02 to 1.12, indicating minimal collinearity and well below commonly used thresholds (i.e., VIF > 2.5, >5, or >10). We ran sensitivity analyses by assessing neighbourhood factors (the density of fruit and vegetable markets, accessibility to jobs) in separate models adjusted for variables selected by partDSA. Additionally, in a sensitivity analysis, we further accounted for factors identified as determinants of UPF in literature and from our unadjusted analyses, but were not selected by partDSA. These factors included household income, maternal ethnicity, maternal education, and child sex.

To further assess the robustness of our results across different classification systems, we replicated our multivariable regression models with highly processed foods (HPF) as the outcome, defined by Poti et al. at the University of North Carolina (UNC)^43^.

Statistical analyses were carried out using the R Studio version 2024.12.1 + 563; R Foundation for Statistical Computing. Figures were generated using the *ggplot2* package, partDSA analyses were conducted using *partDSA* package^39^, and imputation was conducted using the *mice* package^40^ in R Studio.

## Results

### Study population characteristics

A total of 2411 three-year-old children from the CHILD Cohort Study were included in this analysis across four Canadian sites (Table 1). Our study population included a balanced distribution of males (52.4%) and females (47.6%). Most participants came from high SES backgrounds, with a high prevalence of post-secondary education among mothers (78.7%) and fathers (70.5%), and half (54.7%) reporting an annual household income above CAD $100,000. Children were breastfed for an average of 11 months and approximately 45.5% of children had at least one older sibling. Time spent on screen-based technologies averaged 1.7 h per day among children. According to the Canadian Food Environment Dataset (Can-FED), almost half (45.6%) of children resided in neighbourhoods with no accessibility to markets that primarily sell fruit and vegetables. The proportion of those with no accessibility to fast food-restaurants is now reflected in Supplementary Table 2. Additional descriptive characteristics of the study population are shown in Supplementary Table 2.

**Table 1:** Study participants characteristics in the CHILD Cohort Study (n = 2,411)

Parental & Household Factors	Mother	Father
Age, y, mean (SD)	32.5 (4.5)	35.1 (5.5)
Completed post-secondary degree, n (%)	1897 (78.7)	1701 (70.5)
Ethnicity, Caucasian White, n (%)	1797 (74.5)	1816 (75.3)
Western-like dietary pattern score, mean (SD)	-	0.02 (1.0)
UPF intake (% energy), mean (SD)	48.8 (13.6)	-
BMI (kg/m^2^), mean (SD)	25.2 (5.7)	27.4 (4.7)
Family income, CAD, n (%)		
$0 - $59,999	352 (14.6)	
$60,000 - $99,999	577 (23.9)	
$100,000 or over	1318 (54.7)	
Prefer not to say	164 (6.8)	
**Birth and Infancy Factors**		
Sex, male, n (%)	1264 (52.4)	
Gestational age at birth (weeks), mean (SD)	39.2 (1.4)	
Birth weight (g), mean (SD)	3458.0 (477.2)	
Breastfeeding duration (months), mean (SD)	11.1 (6.8)	
**Child Factors at 3-year**		
Age (years), median [IQR]	3.0 [3.0, 3.1]	
Energy intake (kcal/day), median [IQR]	1518.9 [1239.3, 1864.0]	
UPF % energy intake, mean (SD)	45.0 (11.5)	
PF % energy intake, mean (SD)	14.0 (5.3)	
PCI % energy intake, mean (SD)	3.0 (3.1)	
MPF % energy intake, mean (SD)	38.0 (11.0)	
Older siblings, n (%)	1098 (45.5)	
Screentime (hours/day), mean (SD)	1.7 (1.4)	
**Environmental Factors**		
Study site at 3-years, n (%)		
Manitoba	733 (30.4)	
Edmonton	554 (23.0)	
Vancouver	582 (24.1)	
Toronto	542 (22.5)	
Density of fruit and vegetable markets at 3-years, n (%)		
No accessibility	1099 (45.6)	
Lowest to moderate density	746 (30.9)	
Moderate to highest density	556 (23.5)	
Job accessibility via car >median at 3-years, n (%)	1204 (50.0)	
Job accessibility via transit >median at 3-years, n (%)	1204 (50.0)	

Values are mean (SD) for continuous normally distributed variables, median [IQR] for continuous non-normally distributed variables, n (%) for categorical variables. Family, childhood, and environmental characteristics are the pooled values after multiple imputations procedure.

Most of these characteristics were similar among participants who enroled in the CHILD study but did not participate in the follow-up visits and/or had no dietary intake data, therefore not included in this study (*n* = 1043) (Supplementary Table 3). However, among the participants lost to follow-up, there was a lower prevalence of post-secondary education and children had a shorter duration of breastfeeding (Supplementary Table 3).

### UPF consumption across provinces in Canadian preschool children

**Fig. 1:**
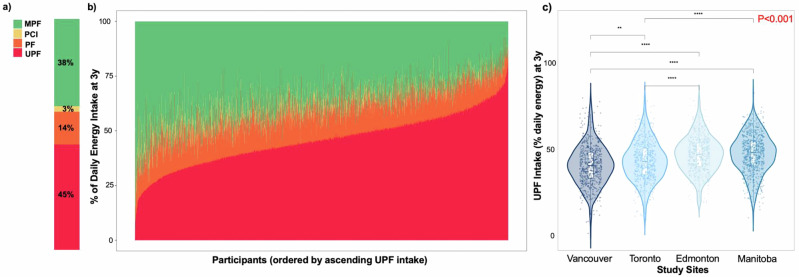
Distribution of NOVA categories at 3 years of age in the CHILD Cohort Study (*n* = 2411). **a** Stacked bar graph showing the mean daily energy intake contribution from NOVA categories across the participants. **b** Stacked bar graph showing the daily energy intake contribution from NOVA categories at the individual level. **c** Violin plot of UPF intake (% daily energy) at 3 years old, across study sites (*N* = 2411). Toronto (*n* = 542), Vancouver (*n* = 582), Edmonton (*n* = 554), Manitoba (*n* = 733). Two-sided *p* < 0.001 according to ANOVA test. Asterisks indicate Tukey HSD–adjusted significance levels (** *p* = 0.003, **** *p* < 0.0001). Source data are available in Supplementary Note. Abbreviations: Minimally processed foods (MPF), processed culinary ingredients (PCI), processed foods (PF), ultra-processed foods (UPF).

On average, UPFs contributed 45.0% of total daily energy intake among three-year-olds (Fig. 1A). As shown in Fig. 1B, UPFs and MPFs made up the majority of energy intake, with UPFs leading in most children. In contrast, processed culinary ingredients (PCI) and processed foods (PF) contributed relatively small and consistent proportions of energy intake across participants. Among all participants, UPF intakes ranged from 1.5% to 83.0% (Fig. 1B and Table 1). There were significant differences in UPF intake across the four study sites (Fig. 1C), with higher intake observed in children from Manitoba and Edmonton compared to those from Toronto and Vancouver.

### Individual correlates of UPF consumption

**Fig. 2:**
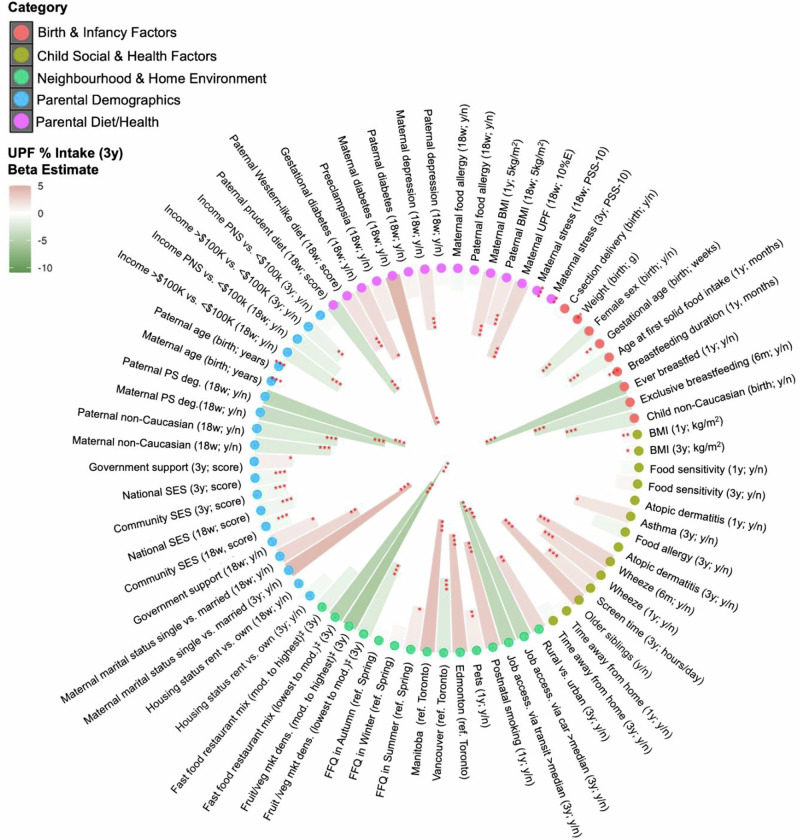
Individual correlates of UPF intake at 3 years of age, spanning across infancy, childhood, parental and environmental factors in the CHILD Cohort Study (*n* = 2411). Beta estimates from unadjusted linear regression models show the association between factors associated with high (red) to low (green) UPF (% daily energy) at 3 years. Factors grouped in five colour coded categories. *Two-sided p-value < 0.05, ** p-value < 0.01, *** p-value < 0.001. ^‡^Density in reference to no access to food outlets. Source data are available in Supplementary Note. Abbreviations: SES socioeconomic status, FFQ food frequency questionnaire, BMI body mass index, mod moderate, fruit/veg mkt fruit and vegetables market, PNS prefer not to say, PS deg post-secondary degree, y/n binary variable comparing yes vs. no, access accessibility, dens density.

Figure 2 shows the results from the unadjusted analyses for identifying the correlates of UPF consumption. Individual correlates of higher child UPF intake at three years of age included factors at infancy (i.e., male sex, younger age at introduction to the first solid foods, shorter breastfeeding duration); social and health factors (i.e., having older siblings, longer exposure to screentime, having wheezing, asthma); neighbourhood and home environment (i.e., lack of food retail accessibility, poor job accessibility, having smokers or pets at home); parental characteristics (i.e., income, no post-secondary education, Caucasian ethnicity, low perceived SES status, maternal single marital status); and parental diet and health factors (high paternal adherence to a Western-like diet pattern, and high maternal UPF intake, maternal diabetes). However, other factors such as time spent away from home (proxy of daycare attendance), having food sensitivity/allergy, and prenatal parental food allergies were not significantly associated with UPF.

### Independent predictors of UPF consumption

All the variables described above (*n* = 65) were further assessed using a Deletion/Substitution/Addition (also known as partitioning using Deletion/Substitution/Addition or partDSA) prediction algorithm, that revealed a total of 11 predictors selected in ≥5% of models. These predictors included maternal and paternal factors (maternal age, prenatal maternal UPF intake, maternal body mass index (BMI), paternal adherence to a Western-like dietary pattern, paternal BMI), child factors (breastfeeding duration, having older siblings, screentime), and neighbourhood factors (fruit and vegetable market density, job accessibility by transit and by car) (Supplementary Fig. 2), with child screen time, maternal UPF intake and job accessibility being the top three identified factors.

**Fig. 3:**
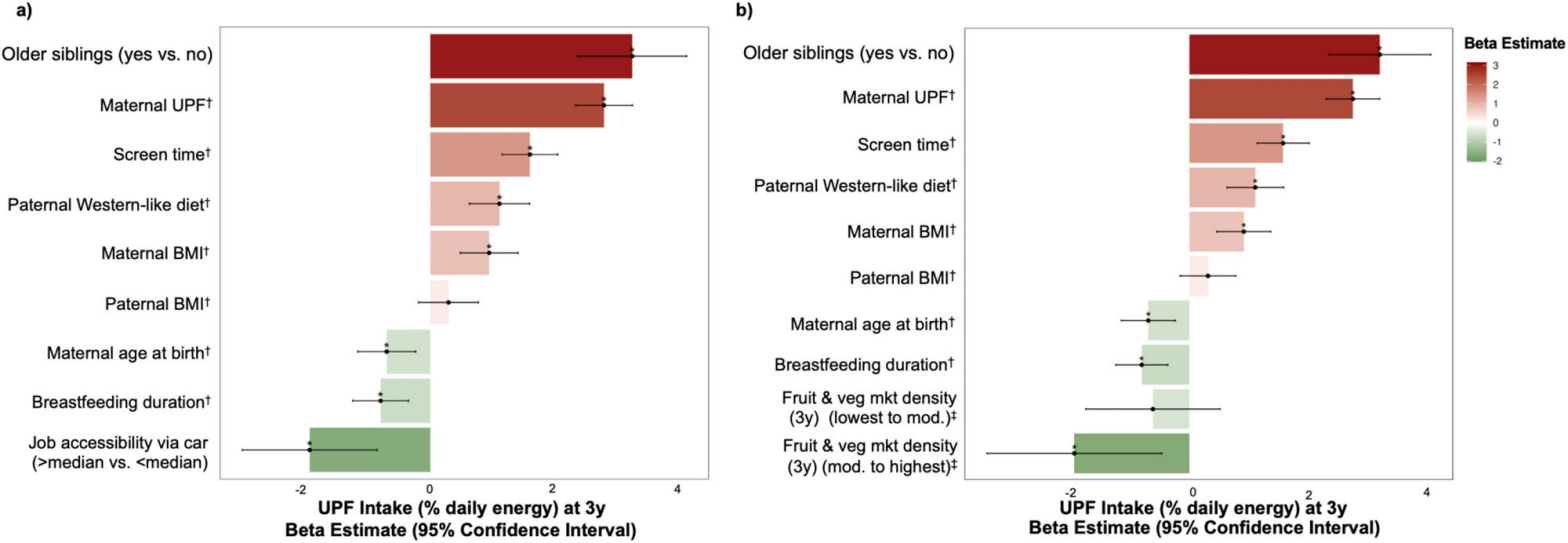
Bar plots of multivariable adjusted linear mixed-effect model showing the independent predictors of UPF intake at 3 years of age in the CHILD Cohort Study (*n* = 2411). Points represent β estimates and error bars indicate 95% confidence intervals for associations between UPF (% daily energy) and variables selected by partDSA, from linear mixed-effects models with study site included as a random effect. Predictors mutually adjusted and selected by partDSA included older siblings (*p* < 0.001), maternal UPF (*p* < 0.001), screen time (*p* < 0.001), paternal Western-like diet (*p* < 0.001), maternal BMI (*p* < 0.001), paternal BMI (*p* = 0.24), maternal age at birth (*p* = 0.003), breastfeeding duration (p < 0.001). Additional factors assessed separately included **a** job accessibility via car (*p* < 0.001) and, **b** neighbourhood density of fruit and vegetable markets (lowest to moderate: *p* = 0.29, moderate to highest: *p* = 0.01). *P-values < 0.05 are two-sided and unadjusted for multiple comparisons. ^†^Continuous variables are expressed per sd-increase. ^‡^Reference to no access to fruit and vegetable markets. Source data are available in Supplementary Note. Abbreviations: UPF ultra-processed foods, mod moderate, BMI Body Mass Index, mrk market.

Family income, education, ethnicity, or child sex were not highly selected by the partDSA prediction algorithm. These factors (maternal education and ethnicity, family income and child sex) have already been identified in the literature as determinants of UPF (% energy intake); therefore, in a sensitivity analysis, we considered them in the multivariable adjusted linear mixed-effect models. Among the partDSA selected predictors, neighbourhood factors, specifically fruit and vegetable market density, and job accessibility by car or transit, were highly correlated (Supplementary Fig. 3). To avoid multicollinearity, we retained the job accessibility variable that was selected more frequently by partDSA (i.e., job accessibility by car) and analysed it separately from fruit and vegetable market density in the multivariable adjusted models (Fig. 3A, B). In a sensitivity analysis, we also examined chain grocery stores, which were not selected by the algorithm, and observed that it was not associated with child UPF intake (Supplementary Fig. 5).

After mutual adjustment of the significant predictors, we found that a variety of family behaviours, childhood characteristics, and features of the built environment remained independently associated with UPF (percentage of daily energy intake) consumption among preschool children (Fig. 3A). For family behaviours, each sd increase in maternal UPF intake and in adherence to paternal Western-like dietary pattern prenatally, were associated with increased UPF intake in children ($\upbeta$= 2.8 higher % daily energy from UPF, [95%CI 2.3, 3.2] and $\upbeta$=1.1 higher % daily energy from UPF, [95%CI 0.6, 1.6], respectively). In addition, higher maternal BMI and younger maternal age at childbirth (per SD-increase) were also associated with increased UPF intake ($\upbeta$= 0.9 higher % daily energy from UPF, [95%CI 0.5, 1.4] and ($\upbeta$= –0.7 % daily energy from UPF, [95%CI –1.2, –0.2], respectively).

Examining child factors, we found that children who were breastfed longer had lower UPF intake at three years of age ($\upbeta$= –0.8 % daily energy from UPF, [95%CI –1.2, –0.4]); while those who had older siblings and spent longer screen time had $\upbeta$= 3.2, [95%CI 2.3, 4.1] and $\upbeta$= 1.6, [95%CI 1.1, 2.0] higher % daily energy from UPF, respectively (Fig. 3A).

Built environment indicators of parental time constraints also emerged as strong predictors. For example, children living in neighbourhoods with greater accessibility to jobs (above median vs. below median) consumed significantly less UPF (β = –1.9 % daily energy from UPF, [95%CI –3.0, –0.9]), suggesting that daily commuting distance, a proxy for time poverty, constrains opportunities for home-prepared meals and healthier food access. In a separate analysis, residing in areas with moderate to high density of fruit and vegetable markets was also associated with lower UPF intake compared to areas with no such markets (β = –2.0 % daily energy from UPF, [95%CI –3.4, –0.5]) (Fig. 3B).

**Fig. 4:**
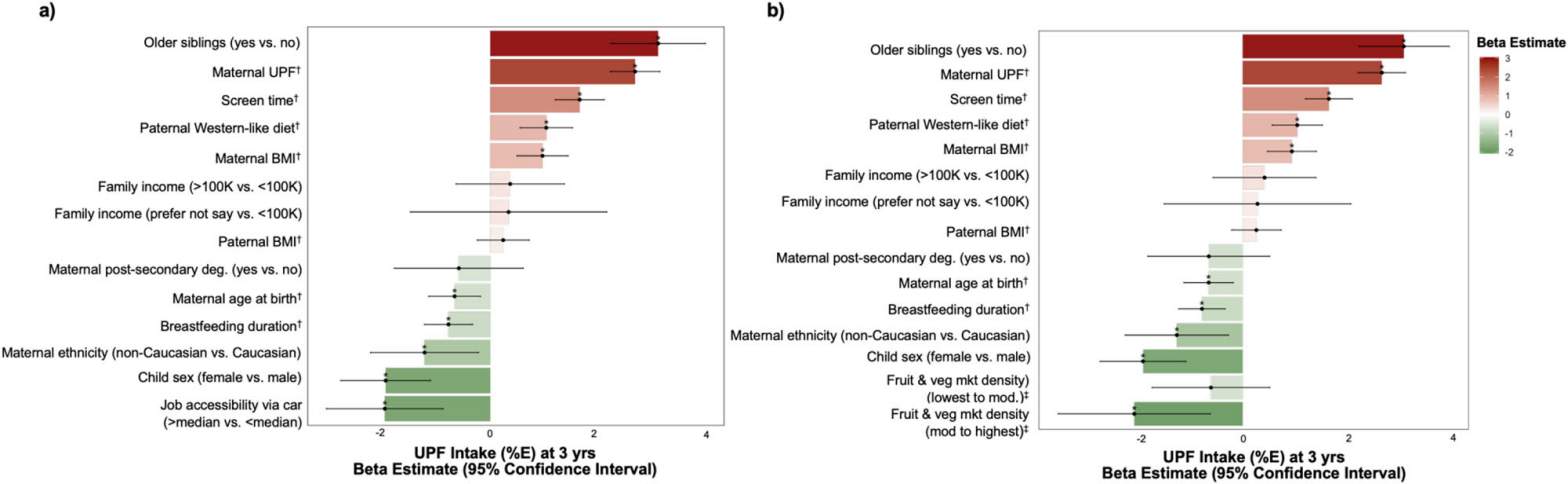
Bar plots of multivariable adjusted linear mixed-effect models showing the independent predictors of UPF intake additionally adjusted for sociodemographic factors at 3 years of age in the CHILD Cohort Study (*n* = 2411). Points represent β estimates and error bars indicate 95% confidence intervals for associations between UPF (% daily energy) and variables selected by partDSA, adjusted for sociodemographic factors from linear mixed-effects models with study site included as a random effect. Predictors mutually adjusted included older siblings (*p* < 0.001), maternal UPF (*p* < 0.001), screen time (*p* < 0.001), paternal Western-like diet (*p* < 0.001), maternal BMI (*p* < 0.001), family income ( > 100 K: *p* = 0.47, prefer not to say: *p* = 0.71), paternal BMI (*p* = 0.33), maternal post-secondary degree (*p* = 0.34), maternal age at birth (*p* = 0.007), breastfeeding duration (*p* < 0.001), maternal non-Caucasian ethnicity (*p* = 0.02), and female child sex (*p* < 0.001). Additional factors assessed separately included **a** job accessibility via car (*p* < 0.001) and, **b** neighbourhood density of fruit and vegetable markets (lowest to moderate: *p* = 0.29, moderate to highest: *p* = 0.005). *P-values < 0.05 are two-sided and unadjusted for multiple comparisons. ^†^Continuous variables are expressed per sd-increase. ^‡^ Reference to no access to fruit and vegetable markets. Source data are available in Supplementary Note. Abbreviations: UPF ultra-processed foods, mod moderate, BMI Body Mass Index, mrk market.

These associations remained relatively unchanged and significantly independent after further adjustment for sociodemographic characteristics not selected by partDSA, including income, maternal ethnicity, and education and child sex (Fig. 4A, B). Among these sociodemographic variables, child sex and maternal ethnicity remained significantly associated with UPF intake, where children of female sex and those whose mothers were identified as non-Caucasian were associated with lower UPF intakes (Fig. 4A, B). Family income and maternal education were not significantly associated with child UPF energy% at preschool age (Fig. 4A, B). Lastly, we ran a sensitivity analysis to determine the robustness of our findings, where we classified highly-processed foods (HPF) based on the University of North Carolina (UNC), which is more reflective of the North American^43^, showing similar trends (Supplementary Fig. 4A, B).

## Discussion

Our study found that UPFs contribute to nearly half (45.0%) of the daily energy intake among Canadian preschoolers, with intakes up to 83.0% for some children. We further found that UPF intake levels differed across provinces in Canada. Using extensive multi-level data from one of the largest national pregnancy cohorts, along with the Canadian Urban Environmental Health Research Consortium (CANUE) and Canadian Food Environment Dataset (CAN-FED), brought together by a robust machine learning method for variable selection, we identified a range of independent predictors spanning parental behaviours, child characteristics, and features of the built environment, from the prenatal to the postnatal period. Therefore, our study addresses a major gap in the comprehensive reporting of independent predictors of UPF intake, particularly among preschoolers.

Our findings reveal the complex and interconnected influences that shape dietary patterns from an early age. First, our results reaffirm the influence of parental dietary patterns, with higher prenatal maternal UPF intake and greater paternal adherence to a Western-like diet pattern being associated with higher UPF intake in children. These results are consistent with broader evidence showing strong concordance between parental and child dietary behaviours. For example, systematic reviews have identified parental dietary intake as one of the most consistently reported determinants of children’s consumption of energy-dense, nutrient-poor foods, including sugar-sweetened beverages, snacks, and foods high in salt, sugar, or fat, many of which are ultra-processed^44,45^. Previous studies further support this association, including evidence from the UK showing that more frequent parental consumption of soda and fast-food was associated with higher UPF intake among preschool-aged children^46^, and from Brazil demonstrating similar parent-child concordance in older children^47^. Together, these findings suggest that parental modelling and household food availability may play central roles in shaping children’s early dietary patterns. It is important to note that the use of different methods to assess parental dietary patterns limits the direct comparability of effect estimates; as a result, they are interpreted mainly based on the direction of effect. Additionally, because parental diet was assessed during the prenatal and preconception period, our findings are consistent with shared family dietary patterns and early food environments that precede child feeding practices. While biological mechanisms such as prenatal flavour exposure or pre-conceptional influences have been proposed in prior literature^48^, our study does not provide direct evidence for such pathways, and these mechanisms remain speculative. Whether parental diet is influenced by fresh foods accessibility and subsequently mediates the child’s UPF intake needs to be further studied.

Among other parental factors, younger maternal age and higher maternal BMI were also associated with increased UPF intake in their children, pointing to underlying challenges such as economic barriers, food literacy, and parental capacity for home-cooked meals^21,49,50^. This is supported by a survey showing that younger parents were more likely to report purchasing prepackaged, processed meals because “they are inexpensive”^50^. Furthermore, lower self-efficacy related to cooking and meal-planning, which are inversely related to child UPF consumption, were additional reasons cited for purchasing these meals^21,50^.

Furthermore, we found longer breastfeeding duration to be an independent predictor of UPF consumption, which agrees with prior literature indicating exclusive breastfeeding for less than four months is associated with higher UPF consumption later in childhood^51,52^, and interventions promoting breastfeeding and healthy complementary feeding practices leading to lower UPF consumption later in childhood^53^. This previous evidence speculated that mothers who breastfed their children longer may consume a less ultra-processed diet and therefore, are more likely to introduce MPFs to their children later on^52^. Furthermore, breastfed infants have been shown to accept vegetable purées more readily than formula-fed infants at the start of weaning^54^. Our findings support ongoing initiatives to promote and support breastfeeding.

Moreover, our results agree with studies relating having older siblings to increased UPF consumption^20,23,55^. This could be explained in part due to the convenient nature of UPF (ready-to-eat, pre-made), higher volume, and longer shelf-life that larger families can benefit from. A study based in the Netherlands found that UPFs were more often promoted in bulk compared to MPF^56^. Similarly, screen time emerged as a significant social determinant of UPF intake, echoing earlier findings^22,49,55^. This can be attributed to digital marketing of UPFs, which is heavily targeted at children and influences parental purchasing decisions^57,58^. Higher screen time has also been linked to lower fruit and vegetable intake and increased snacking on energy-dense foods among children, likely due to their convenience and rapid consumption^59^.

Importantly, our findings extend beyond household-level predictors to highlight built environment predictors of childhood diet. Children living in neighbourhoods with low accessibility to jobs (by car or transit), an indicator of parental time poverty and reduced flexibility, consumed significantly more UPF on average. This is supported by a Canadian study that identified ‘time’ as the most reported barrier for meal preparation among parents, especially for those with full-time employment status^60^. Similarly, lower density of fruit and vegetable markets was associated with greater UPF consumption, which aligns with findings from the SENDO project in Spain where children from families with access to shopping in specialised stores (i.e., local fresh food markets) had higher intakes of MPFs, compared to children from families shopping in hypermarkets (i.e., modern retail outlets)^61^. These findings suggest that commute times, spatial food retail patterns, and local infrastructure all interact with dietary behaviours, reinforcing the need for integrated planning across (public) health and urban sectors^60,61^.

Lastly, in our study, sociodemographic characteristics (i.e., education and income) were not among the most highly selected predictors, and sensitivity analyses adjusting for these variables did not identify them as significant independent determinants of child UPF intake. This is in contrast with previous research that has identified sociodemographic characteristics as contributors of UPF intakes^16,18^. This may be because other studies did not additionally account for other factors such as parental diet and food environment, and instead focus on sociodemographic characteristics alone^16,18^. Another possible explanation is that the CHILD Cohort Study includes a slightly more socioeconomically advantaged population. Approximately 70% of parents reported having completed post-secondary education, and nearly half reported an annual household income above CAD $100,000. Although direct comparisons with national estimates are limited by differences in measurement and sampling, these values are slightly higher than Canadian population benchmarks during a similar period, when approximately 52% of adults aged 25–64 had post-secondary education^62^ and the median before-tax household income was $84,200^63^ However, despite this, UPF intake in preschoolers participating in the CHILD study was similar to the national average^3^. After further analysis, we also found that maternal ethnicity and child sex, although not selected by the prediction model, remained significant in the multivariable-adjusted mixed-effect models, which aligns with previous research^16,18^.

Our findings demonstrate how early-life diet is embedded within broader systems of family life, labour markets, and urban design. This moves the discussion beyond the nutrition field toward cross-sectoral strategies, such as transportation planning, parental leave reform, and digital marketing regulation. By integrating these perspectives, our study provides a framework for understanding the structural origins of dietary behaviours, aligning with the current view of tackling obesogenic environments^64^. These might include providing incentives to small retailers to stock up on fresh foods in underserved areas as done in the New Jersey’s Healthy Small Food Retailer Act^65^. Given the influence of parental time poverty, reflected in lower job accessibility, parental leave reforms or hybrid working models (e.g., working certain days from home) may help reduce time constraints on food preparation. Food literacy programmes such as school-based gardening, cooking, and nutrition education intervention also serve as key strategies as they have been demonstrated to effectively reduce children’s UPF consumption and increase their whole foods consumption^66^. The identification of screentime as an independent predictor also underscores the need for improved regulation of digital food marketing directed at young children^64^. Moreover, given ongoing consumer confusion in identifying UPFs^67^, efforts to improve nutrition literacy, particularly among parents, may also help reduce UPF purchases.

Our findings hold important implications for achieving global policy targets such as the United Nations Sustainable Development Goals (SDG)^68^. Reducing UPF consumption in early life aligns with SDG 3 (Good Health and Well-being) by mitigating early risk factors for noncommunicable diseases, and SDG 2 (Zero Hunger) by promoting access to nutritious foods during critical developmental periods. Furthermore, our identification of environmental/neighbourhood-level factors, such as access to jobs and fresh food markets, supports SDG 11 (Sustainable Cities and Communities), emphasising the role of equitable urban design in enabling healthy behaviours. Focusing on the factors that determine early UPF consumption requires coordinated action across sectors and aligns with global efforts to improve population health and meet the SDG.

One strength of our study includes the longitudinal data from parental behaviours, child characteristics, and features of the built environment, extending beyond sociodemographic characteristics. Furthermore, employing a widely used machine learning approach for variable selection, improved our prediction accuracy via cross-validation without requiring strict parametric assumptions^39,42^. Sociodemographic variables such as household income and parental education were not identified as independent predictors of children’s UPF intake in this study. This finding should be interpreted in the context of the study population, which represents a relatively socioeconomically advantaged subset of Canadian families. Limited variability in SES may have reduced our ability to detect independent associations with these factors. Nevertheless, the high levels of UPF intake observed even in this relatively advantaged cohort highlight the widespread nature of UPF exposure and point to influences beyond traditional sociodemographic factors, including parental behaviours and neighbourhood environments. Future studies should explore how these findings generalise to more diverse populations and examine longitudinal dietary trajectories in response to policy and environmental changes. Additionally, FFQs do not capture details such as individual ingredients or food preparation methods (i.e., homemade, commercially prepared), so mapping FFQ items into NOVA groups may require assumptions made. As in other cohort studies^69^, when mapping FFQ items in NOVA groups, we followed a conservative classification approach. Although some misclassification is possible, our UPF intake estimates are comparable to national estimates from 24-hour recalls, and recent studies show acceptable agreement between standard and NOVA-specific FFQs^70^. More detailed dietary assessment methods, such as image or barcode-based tools, may further improve UPF classification accuracy in future studies. Lastly, another limitation is the use of different dietary assessment tools for mothers and fathers. Maternal diet was assessed using a comprehensive, validated food frequency questionnaire that allowed item-level classification according to the NOVA system, whereas paternal diet was captured using a brief food group–based screener. This difference may introduce non-comparability and measurement error, potentially attenuating associations between paternal diet and maternal estimates. As a result, paternal UPF intake could not be directly quantified, and paternal diet was instead characterised using data-driven dietary patterns. While this approach has been widely used in epidemiological studies, findings related to maternal and paternal diet should be interpreted with consideration of these methodological differences.

In conclusion, this study identified a wide range of independent predictors of UPF consumption among Canadian preschoolers, reflecting the convergence of family behaviours and the built environment. These findings reinforce the need for systems-level public health and policy interventions to promote healthier food environments and behaviours. Reducing UPF intake in early life requires action across multiple systems, such as supporting families through social and employment policies, reshaping urban environments to facilitate fresh food access, and regulating the digital marketing ecosystem. Our study illustrates how integrating machine learning with cohort and environmental data can reveal these converging determinants, advancing a systems-based approach to child health. As we move forward in food system transformations, evidence from our study can help inform integrated strategies that span urban planning, nutrition policy, and family support, advancing the 2030 Agenda for Sustainable Development countries.

## Supplementary information

Supplementary Materials

Description of Additional Supplementary files

Supplement Data

## Data Availability

Data described in the manuscript will be made available upon request pending approval from CHILD’s Access and Publication Committee and the CHILD Study National Coordinating Center. Researchers interested in collaborating on a project and accessing CHILD Cohort Study data should contact the Study’s National Coordinating Center (NCC) to discuss their needs before initiating a formal request. To contact the NCC, please email child@mcmaster.ca. A list of variables available in the CHILD Cohort Study is available at https://childstudy.ca/for-researchers/study-data/. More information about data access for the CHILD Cohort Study can be found at https://childstudy.ca/for-researchers/data-access/. Source data for figures are provided as Supplementary Data.

## Code availability

The analytic code can be made available upon request.
