# Supplementary material for: Multilevel predictors of ultra-processed food intake in Canadian preschoolers
Source: Commun Med (Lond). 2026 Mar 6;6:212. doi: 10.1038/s43856-026-01473-1 (PMC13068883; doi:10.1038/s43856-026-01473-1)
Supplement: Supplementary file 1 — Supplementary Materials [file 43856_2026_1473_MOESM1_ESM.pdf]

SUPPLEMENTARY MATERIAL

Multilevel Predictors of Ultra-Processed Food Intake in Canadian Preschoolers

Sara Mousavi[1†], Zheng Hao Chen[1†], Zihang Lu[2], Susana Santos[3], Mary L'Abbe[1], Meghan B. Azad[4], Piushkumar J Mandhane[5], Theo J Moraes[6], Padmaja Subbarao[6,8-10] Stuart E Turvey[7], Jeffrey R Brook[10], and Kozeta Miliku[1,9]

**Supplementary Figure 1**	Flow chart of study population.
**Supplementary Figure 2**	Variable selection using partitioning Deletion Substitution Addition (partDSA) algorithm.
**Supplementary Figure 3**	Heatmap of correlations among neighborhood environment variables selected by partDSA.
**Supplementary Figure 4**	Bar plots of multivariable adjusted linear mixed-effect models of mutually adjusted predictors with highly processed foods (HPF) as the outcome (n=2,411).
**Supplementary Figure 5**	Bar plots of multivariable adjusted linear mixed-effect model showing the independent predictors of UPF intake adjusted for density of chain grocery stores (n=2,411).
**Supplementary Table 1**	Descriptive characteristics of the original and multiple imputed data in the CHILD Cohort Study (n=2,411).
**Supplementary Table 2**	Additional descriptive characteristics of study population (n=2,411)
**Supplementary Table 3**	Non-response analysis of participants included in this study (n=2,411) vs participants enrolled in the CHILD Cohort Study but not having diet data at the three-year visit (n=1,043).

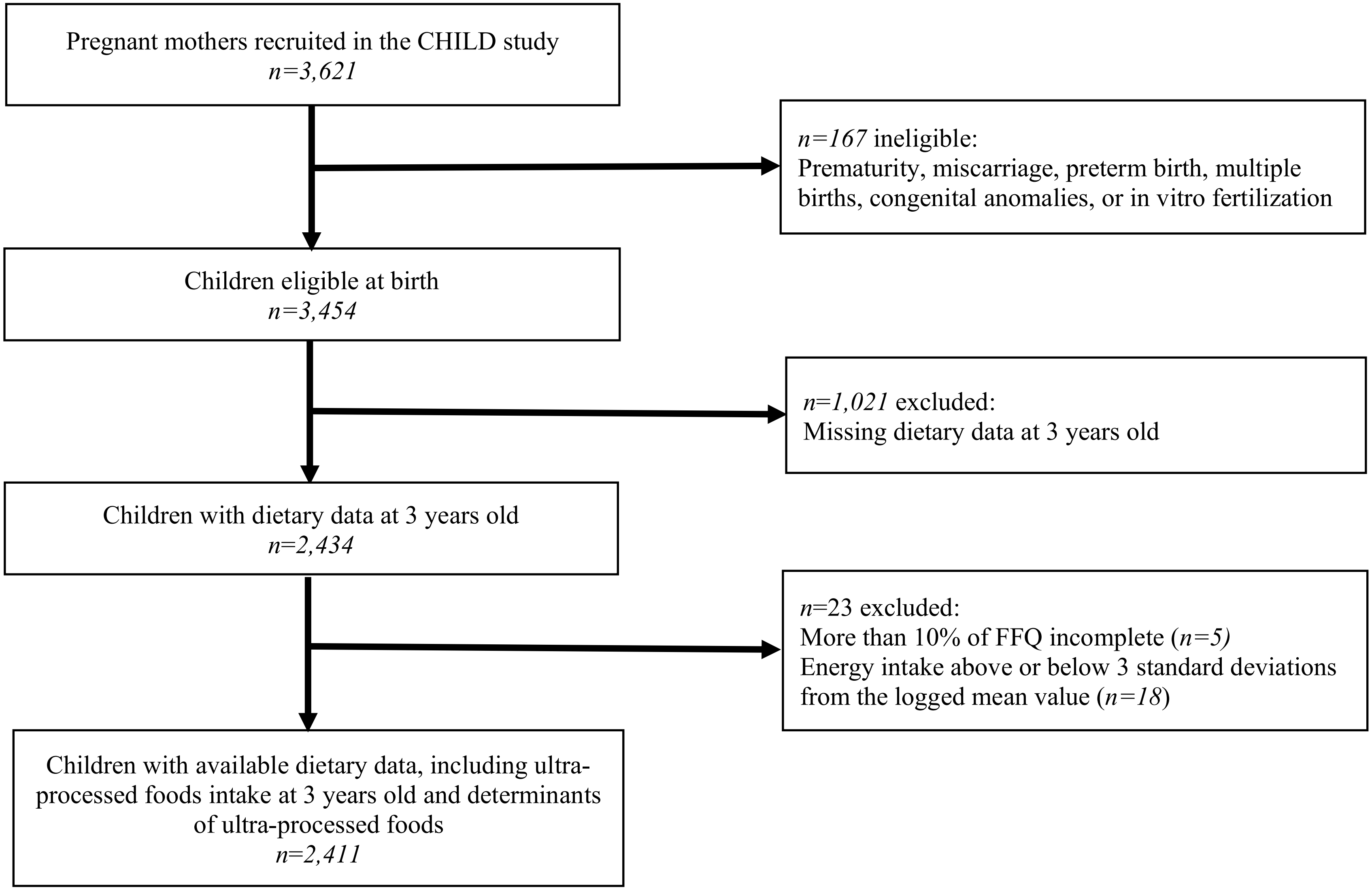

Supplementary Figure 1. Flow chart of the study population.

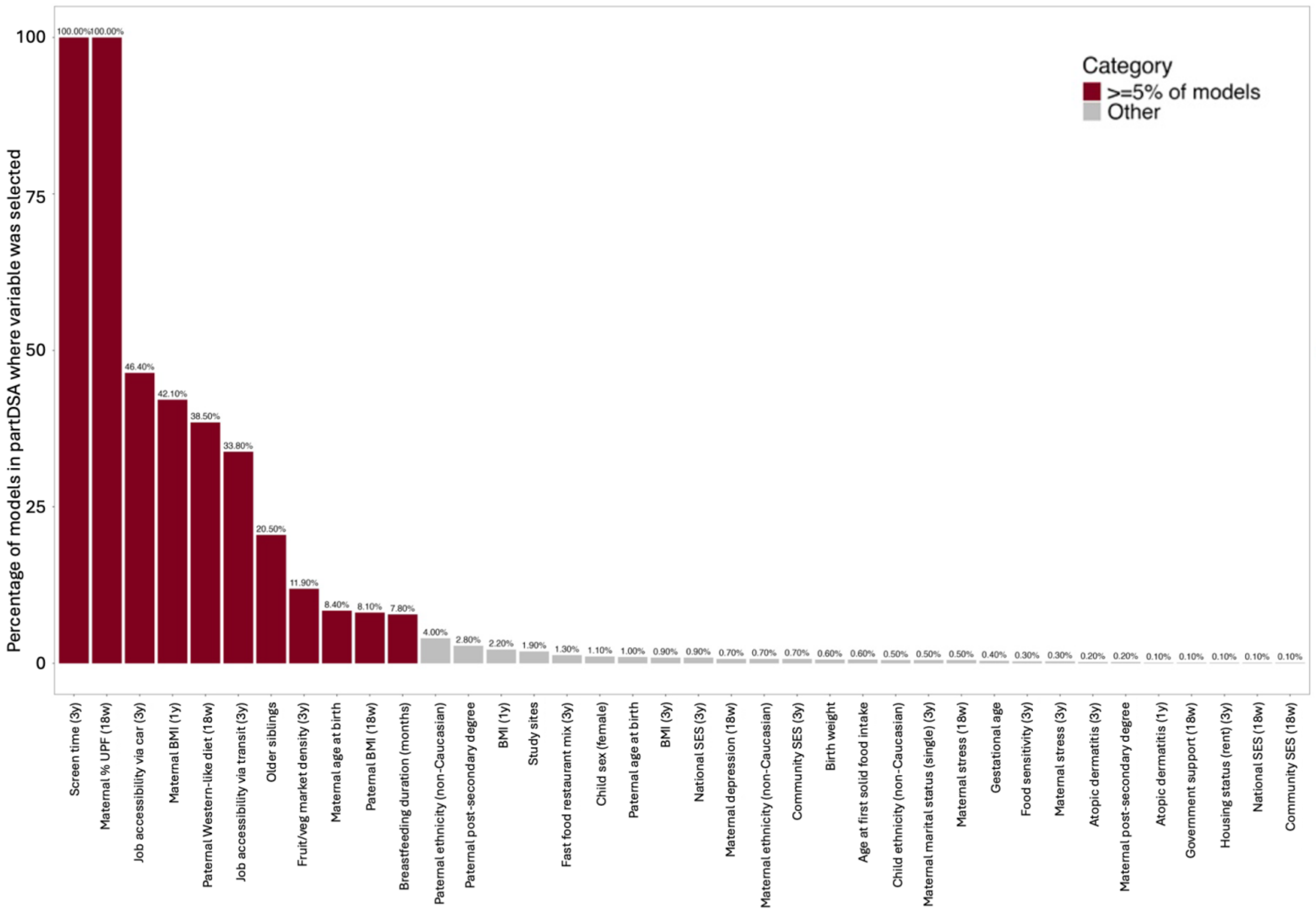

Supplementary Figure 2. Variable selection using partitioning Deletion Substitution Addition (partDSA) algorithm. The number of models in which each variable was selected by partDSA out of 100 iterations was averaged across 10 multiply imputed datasets. Variables in at least 5% of the models (in red) were included in further analyses.

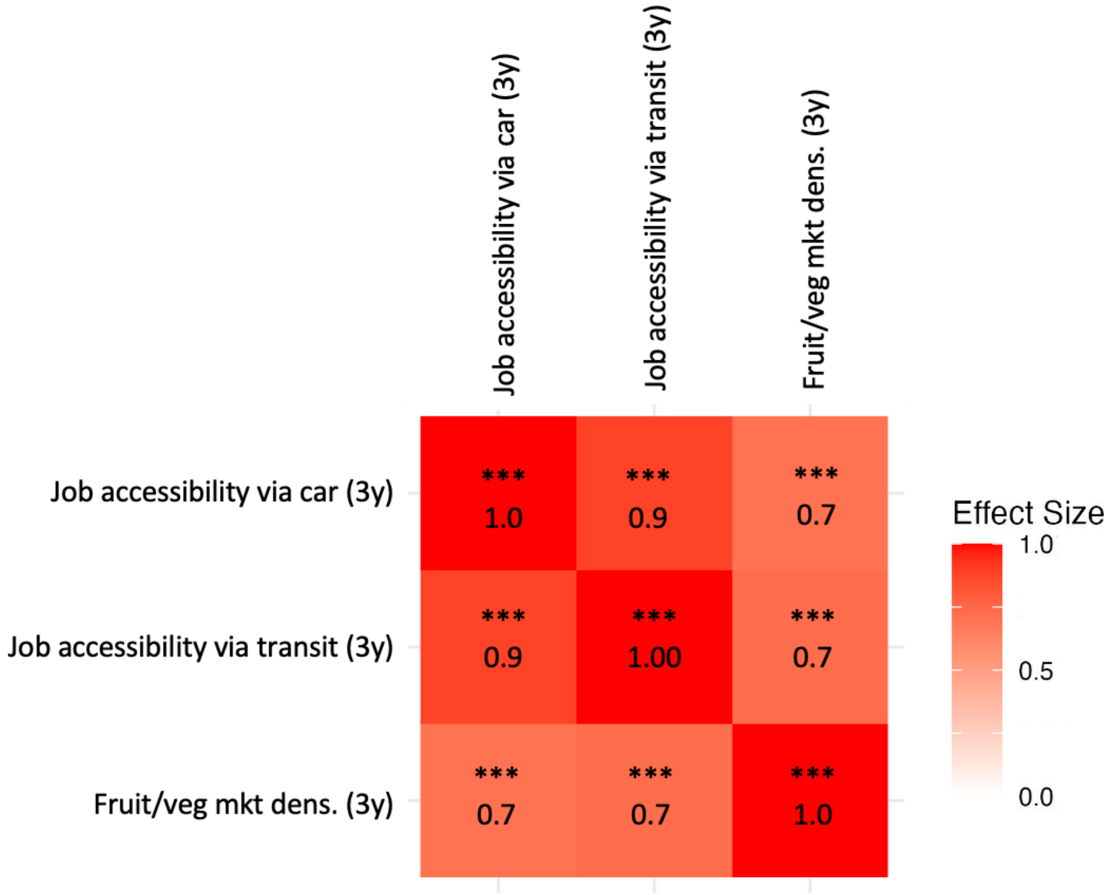

Supplementary Figure 3. Heatmap of correlations among neighborhood environment variables selected by partDSA. Effect sizes correspond to spearman coefficient correlations. ***p<0.001.

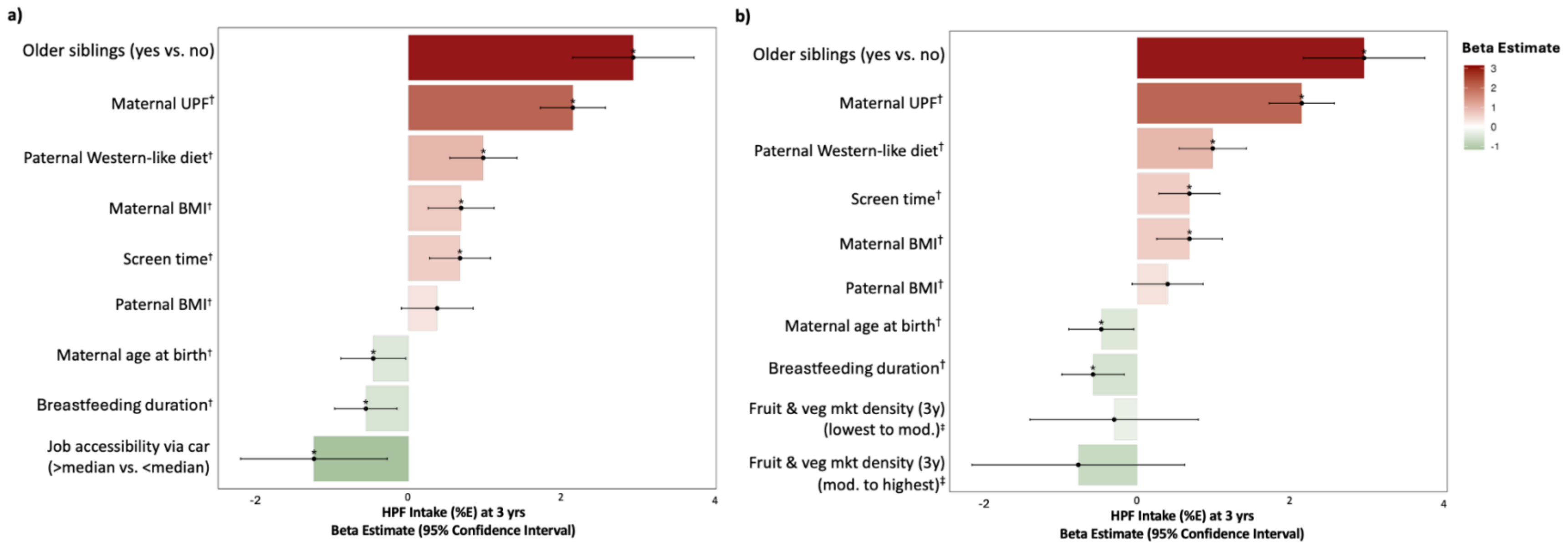

Supplementary Figure 4. Bar plots of multivariable adjusted linear mixed-effect models of mutually adjusted predictors with highly processed foods (HPF) as the outcome in the CHILD Cohort Study (n=2,411). Points represent β estimates and error bars indicate 95% confidence intervals for associations between HPF (% daily energy) and variables selected by partDSA, from linear mixed-effects models with study site included as a random effect. Predictors mutually adjusted and selected by partDSA included older siblings (p<0.001), maternal UPF (p<0.001), screen time (p<0.001), paternal Western-like diet (p<0.001), maternal BMI (p=0.002), paternal BMI (p=0.12), maternal age at birth (p = 0.03), breastfeeding duration (p=0.007). Additional factors assessed separately included **a)** job accessibility via car (p=0.01) and, **b)** neighborhood density of fruit and vegetable markets (lowest to moderate: p = 0.59, moderate to highest: p = 0.28). *P-values <0.05 are two-sided and unadjusted for multiple comparisons. †Continuous variables are expressed per sd-increase. ‡reference to no access to fruit and vegetable markets. Abbreviations: UPF – ultra-processed foods, mod – moderate, BMI – Body Mass Index, mrk – market.

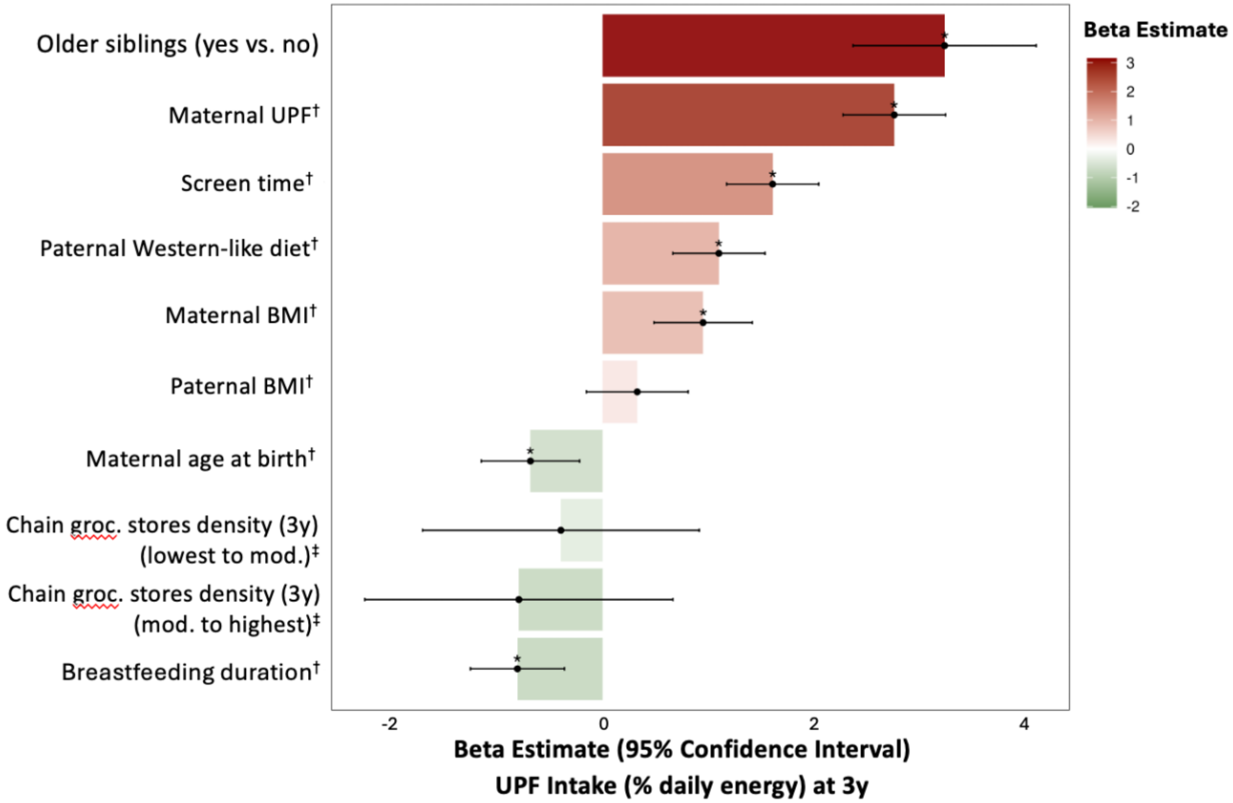

Supplementary Figure 5. Bar plots of multivariable adjusted linear mixed-effect model showing the independent predictors of UPF intake adjusted for density of chain grocery stores in the neighbourhood at 3 years of age in the CHILD Cohort Study (n=2,411). Points represent β estimates and error bars indicate 95% confidence intervals for associations between UPF (% daily energy) and variables selected by partDSA, adjusted for density of chain grocery stores from linear mixed-effects models with study site included as a random effect. *Two-sided p-value <0.05, †Continuous variables are expressed per sd-increase. ‡ reference to no access to chain grocery stores. Abbreviations: UPF- ultra-processed foods, mod - moderate, BMI – Body Mass Index, groc – grocery.

Supplementary Table 1. Descriptive characteristics of the original and imputed data in the CHILD Cohort Study (n=2,411).

	Original		Imputed	
**Prenatal Parental Factors**	**Mother**	**Father**	**Mother**	**Father**
Age, y, mean (SD)	32.5 (4.5)	35.1 (5.4)	32.5 (4.5)	35.1 (5.5)
Missing	0 (0.0)	190 (7.9)	0 (0.0)	0 (0.0)
Completed post-secondary degree, n (%)	1858 (77.1)	1656 (68.7)	1897 (78.7)	1701 (70.5)
Missing	56 (2.3)	75(3.1)	0 (0.0)	0 (0.0)
Ethnicity, Caucasian, n (%)	1789 (74.2)	1813 (75.2)	1797 (74.5)	1816 (75.3)
Missing	9 (0.4)	4 (0.2)	0 (0.0)	0 (0.0)
Married marital status, n (%)	2263 (93.9)	-	2294 (95.2)	-
Missing	33 (1.4)	-	0 (0.0)	-
Western-like dietary pattern, mean (SD)	-	0.0 (1.0)	-	0.02 (1.0)
Missing	-	371 (15.4)	-	0 (0.0)
UPF intake, mean (SD)	48.9 (13.5)	-	48.8 (13.5)	-
Missing	152 (6.3)	-	0 (0.0)	-
BMI (kg/m^2), mean (SD)	25.2 (5.7)	27.4 (4.7)	25.2 (5.7)	27.4 (4.7)
Missing	69 (2.9)	481 (20.0)	0 (0.0)	0 (0.0)
**Household Factors at 3-year**				
Family income, n (%)				
<$100,000	834 (34.6)		929 (38.5)	
≥$100,000	1201 (49.8)		1318 (54.7)	
Prefer not to say	150 (6.2)		164 (6.8)	
Missing	411 (17.0)		0 (0.0)	
Housing status, own, n (%)	1793 (74.4)		1962 (81.4)	
Missing	216 (9.0)		0 (0.0)	
Government sources contribute to income, n (%)	1174 (48.7)		1326 (55.0)	
Missing	268 (11.1)		0 (0.0)	
**Birth and Infancy**				
C-section delivery, n (%)	594 (24.6)		603 (25.0)	
Missing	33 (1.4)		0 (0.0)	
Sex, male, n (%)	1264 (52.4)		1264 (52.4)	

Missing	0 (0.0)	0 (0.0)
Gestational age at birth (weeks), mean (SD)	39.2 (1.4)	39.2 (1.4)
Missing	35 (1.5)	0 (0.0)
Birth weight (g), mean (SD)	3458.2 (476.7)	3458.0 (477.2)
Missing	48 (2.0)	0 (0.0)
Breastfeeding duration (months), mean (SD)	11.1 (6.8)	11.1 (6.8)
Missing	8 (0.3)	0 (0.0)
**Child Health at 3-year**		
Energy intake (kcal/day), median [IQR]	1518.9 [1239.3, 1864.0]	1518.9 [1239.3, 1864.0]
Missing	0 (0)	0 (0.0)
BMI (kg/m^2), mean (SD)	16.3 (1.4)	16.3 (1.4)
Missing	125 (5.2)	0 (0.0)
Food allergy, n (%)	219 (9.1)	230 (9.5)
Missing	98 (4.1)	0 (0.0)
Atopic dermatitis, n (%)	430 (17.8)	453 (18.8)
Missing	101 (4.2)	0 (0.0)
Asthma, n (%)	290 (12.0)	301 (12.5)
Missing	97 (4.0)	0 (0.0)
**Child Social Factors**		
Older siblings, n (%)	1098 (45.5)	1098 (45.5)
Missing	0 (0.0)	0 (0.0)
Screentime at 3-year (hours/day), mean (SD)	1.7 (1.4)	1.7 (1.4)
Missing	70 (2.9)	0 (0.0)
Day care attendance at 3-year (proxy time away from home), n (%)	1309 (54.3)	1404 (58.2)
Missing	173 (7.2)	0 (0.0)
**Environmental Factors**		
Smokers at home at prenatal or 1-year, n (%)	382 (15.8)	384 (15.9)
Missing	8 (0.3)	0 (0.0)
Pets at home at 1-year, n (%)	934 (38.7)	1054 (43.7)
Missing	285 (11.8)	0 (0.0)

Study site at 3-years, n (%)		
Manitoba	733 (30.4)	733 (30.4)
Edmonton	554 (23.0)	554 (23.0)
Vancouver	582 (24.1)	582 (24.1)
Toronto	542 (22.5)	542 (22.5)
Missing	0 (0.0)	0 (0.0)
Season at 3-years, n (%)		
Spring	653 (27.1)	654 (27.1)
Summer	615 (25.5)	616 (25.5)
Winter	570 (23.5)	571 (23.7)
Autumn	569 (23.6)	570 (23.6)
Missing	4 (0.2)	0 (0.0)
Density of fruit and vegetable markets at 3-years, n(%)		
No accessibility	901 (37.4)	1099 (45.6)
Lowest to moderate density	660 (27.4)	746 (30.9)
Moderate to highest density	524 (21.7)	556 (23.5)
Missing	326 (13.5)	0 (0.0)
Density of fast-food restaurant mix at 3-years, n (%)		
No accessibility	196 (8.1)	227 (9.4)
Lowest to moderate density	1369 (56.8)	1542 (63.9)
Moderate to highest density	520 (21.6)	642 (26.6)
Missing	326 (13.5)	0 (0.0)
Job accessibility via car >median at 3-years, n (%)	1076 (44.6)	1204 (50.0)
Missing	415 (17.3)	0 (0.0)
Job accessibility via transit >median at 3-years, n (%)	1062 (44.0)	1204 (50.0)
Missing	415 (17.3)	0 (0.0)

Values are mean (SD) for continuous normally distributed variables, median [IQR] for continuous non-normally distributed variables, n (%) for categorical variables. Family, childhood, and environmental characteristics are the pooled values after multiple imputations procedure.

Supplementary Table 2. Additional descriptive characteristics of study population (n=2,411)

**Parental & Household Factors**	**Mother**	**Father**
Married marital status, married, n (%)	2294 (5.2)	-
Housing status, own, n (%)	1962 (81.4)	
Government sources contribute to income, n (%)	1326 (55.0)	
**Birth and Infancy Factors**		
C-section delivery, n (%)	603 (25.0)	
**Child Factors at 3-year**		
BMI (kg/m^2), mean (SD)	16.3 (1.4)	
Food allergy, n (%)	230 (9.5)	
Atopic dermatitis, n (%)	453 (18.8)	
Asthma, n (%)	301 (12.5)	
Time away from home (day care attendance), n (%)	1404 (58.2)	
**Environmental Factors**		
Smokers at home at prenatal or 1-year, n (%)	384 (15.9)	
Pets at home at 1-year, n (%)	1054 (43.7)	
Season of diet assessment at 3-years, n (%)		
Spring	654 (27.1)	
Summer	616 (25.6)	
Winter	571 (23.7)	
Autumn	570 (23.6)	
Density of fast-food restaurant mix at 3-years, n (%)		
No accessibility	227 (9.4)	
Lowest to moderate density	1542 (63.9)	
Moderate to highest density	642 (26.6)	

Values are mean (SD) for continuous normally distributed variables, median [IQR] for continuous non-normally distributed variables, n (%) for categorical variables. Family, childhood, and environmental characteristics are the pooled values after multiple imputations procedure.

Supplementary Table 3. Non-response analysis of participants included in this study (n=2,411) vs participants enrolled in the CHILD Cohort Study but not having diet data at the three-year visit (n=1,043).

	**Participants included in analysis (n=2411)**		**Participants excluded from analysis (n=1043)**	
**Parental Factors**	**Mother**	**Father**	**Mother**	**Father**
Age, y, mean (SD)	32.5 (4.5)	35.1 (5.4)	32.1 (5.3)†	34.3 (5.6)
Missing	0 (0.0)	190 (7.9)	205 (19.7)	659 (63.2)
Completed post-secondary degree, n (%)	1858 (77.1)	1656 (68.7)	535 (51.3)†	454 (46.4)†
Missing	56 (2.3)	75 (3.1)	266 (25.5)	276 (26.5)
Ethnicity, Caucasian White, n (%)	1789 (74.2)	1813 (75.2)	550 (52.7)†	561 (53.8)†
Missing	9 (0.4)	4 (0.2)	233 (22.3)	225 (21.6)
Married marital status, married, n (%)	2263 (93.9)	-	701 (67.2)†	-
Missing	33 (1.4)	-	266 (25.5)	-
Western-like dietary pattern score, mean (SD)	-	0.0 (1.0)	-	0.0 (1.1)
Missing	-	371 (15.4)	-	442 (42.4)
UPF intake, mean (SD)	48.9 (13.5)	-	49.2 (14.8)	-
Missing	152 (6.3)	-	330 (31.6)	-
BMI (kg/m^2), mean (SD)	25.2 (5.7)	27.4 (4.7)	26.2 (6.6)†	27.5 (4.5)
Missing	69 (2.9)	481 (20.0)	427 (40.9)	658 (63.1)
**Household Factors**				
Family income, n (%)				
<$100,000	834 (34.6)		23 (2.2)†	
≥$100,000	1201 (49.8)		13 (1.2)†	
Prefer not to say	150 (6.2)		5 (0.5)†	
Missing	411 (17)		1002 (96.1)	
Housing status, own, n (%)	1793 (74.4)		30 (2.9)†	
Missing	216 (9.0)		1002 (96.1)	
Government sources contribute to income, n (%)	1174 (48.7)		96 (9.2)†	
Missing	268 (11.1)		931 (89.3)	

**Birth and Infancy**		
C-section delivery, n (%)	594 (24.6)	219 (21.0)
Missing	33 (1.4)	205 (19.7)
Sex, male, n (%)	1264 (52.4)	552 (52.9)
Missing	0 (0.0)	0 (0.0)
Gestational age at birth (weeks), mean (SD)	39.2 (1.4)	39.2 (1.4)
Missing	35 (1.5)	26 (2.5)
Birth weight (g), mean (SD)	3458.2 (476.7)	3413.5 (493.4)†
Missing	48 (2.0)	38 (3.6)
Breastfeeding duration (months), median [IQR]	11.1 (6.8)	8.1 (6.3)†
Missing	8 (0.3)	289 (27.7)
**Child Health at 3-year**		
Energy intake (kcal/day), median [IQR]	1518.9 [1239.3, 1864.0]	-
Missing	0 (0.0)	-
BMI (kg/m^2), mean (SD)	16.3 (1.4)	16.4 (1.5)
Missing	125 (5.2)	474 (45.4)
Food allergy, n (%)	219 (9.1)	44 (4.2)
Missing	98 (4.1)	638 (61.2)
Atopic dermatitis, n (%)	430 (17.8)	74 (7.1)
Missing	101 (4.2)	636 (61.0)
Asthma, n (%)	290 (12.0)	97 (4.0)†
Missing	97 (4.0)	636 (61.0)
**Child Social Factors**		
Older siblings, n (%)	1098 (45.5)	414 (39.7)
Missing	0 (0.0)	192 (18.4)
Screentime at 3-year (hours/day), mean (SD)	1.7 (1.4)	1.8 (1.4)
Missing	70 (2.9)	935 (89.6)
Day care attendance (proxy time away from home) at 3-year, n (%)	1309 (54.3)	18 (1.7)
Missing	173 (7.2)	191 (18.3)
**Environmental Factors**		
Smokers at home at 1-year, n (%)	382 (15.8)	198 (19.0)†

Missing	8 (0.3)	243 (23.3)
Pets at home at 1-year, n (%)	934 (38.7)	192 (18.4)
Missing	285 (11.8)	587 (56.3)
Study site at 3-years, n (%)		
Edmonton	554 (23.0)	181 (17.4)†
Toronto	542 (22.5)	214 (20.5)†
Manitoba	733 (30.4)	255 (24.4)†
Vancouver	582 (24.1)	131 (12.6)†
Missing	0 (0.0)	262 (25.1)
Season of diet assessment at 3-years, n (%)		
Spring	653 (27.1)	4 (0.4)
Summer	615 (25.5)	6 (0.6)
Autumn	569 (23.6)	9 (0.9)
Winter	570 (23.6)	7 (0.7)
Missing	4 (0.2)	1017 (97.5)
Density of fruit and vegetable markets at 3-years, n (%)		
No accessibility	901 (37.4)	257 (24.6)
Lowest to moderate density	660 (27.4)	170 (16.3)
Moderate to highest density	524 (21.7)	122 (11.7)
Missing	326 (13.5)	494 (47.4)
Density of fast-food restaurant mix at 3-years, n (%)		
No accessibility	196 (8.1)	40 (3.8)
Lowest to moderate density	1369 (56.8)	303 (29.1)
Moderate to highest density	520 (21.6)	206 (19.8)
Missing	326 (13.5)	494 (47.4)
Job accessibility via car >median at 3-years, n (%)	1076 (44.6)	30 (2.9)
Missing	415 (17.2)	981 (94.1)
Job accessibility via transit >median at 3-years, n (%)	1062 (44.0)	32 (3.1)
Missing	415 (17.2)	981 (94.1)

Values are frequency counts and percentages (%) for categorical variables, means and standard deviation (SD) for continuous variables with a normal distribution, or medians and interquartile range (IQR – 25th and 75th percentiles) for continuous variables with a skewed distribution based on non-imputed data. Non-responsive analysis was done between participants included in the

analysis (n=2411) and participants enrolled in the study but excluded due to loss to follow-up or having no dietary intake data (n=1043) using an independent sample t-test for normally distributed variables, a Mann–Whitney U test for non-normally distributed variables, and chi-square test for categorical variables. †p-value < 0.05
